# Validation and Psychometric Evaluation of the Italian Version of the Bergen–Yale Sex Addiction Scale

**DOI:** 10.1007/s11469-021-00597-w

**Published:** 2021-08-09

**Authors:** Paolo Soraci, Francesco M. Melchiori, Elena Del Fante, Roberto Melchiori, Eleonora Guaitoli, Fulvia Lagattolla, Grazia Parente, Enrico Bonanno, Laura Norbiato, Roberta Cimaglia, Lorenzo Campedelli, Francesco Antonio Abbiati, Ambra Ferrari, Mark D. Griffiths

**Affiliations:** 1 Associazione Psicoterapia Cognitivo Comportamentale Di Gruppo, Rome, Italy; 2 Faculty of Psychology, University Niccolò Cusano, Rome, Italy; 3 Department of Psychology, Università degli Studi di Torino–UNITO, Turin, Italy; 4 Faculty of Educational Sciences, University Niccolò Cusano, Rome, Italy; 5 Department of General Surgery, Perrino Hospital Brindisi, Brindisi, Italy; 6 Servizio Di Psiconcologia, IRCCS Istituto Tumori “Giovanni Paolo II” Di Bari, Bari, Italy; 7 Associazione Matrice Orientamento E Formazione Onlus, Bari, Italy; 8 Department of Environmental Research and Innovation, Luxembourg Institute of Science and Technology, Belvaux, Luxembourg; 9 Associazione La Valigia Rossa, Rome, Italy; 10 Istituto Romano Di Psicoterapia Psicodinamica Integrata, Rome, Italy; 11 Department of Psychology, Niccolò Cusano University, Rome, Italy; 12 Department of Human Science for Education, Università Degli Studi Di Milano, Bicocca, Milan Italy; 13 International Gaming Unit, Psychology Department, Nottingham Trent University, 50 Shakespeare Street, Nottingham, NG1 4FQ UK

**Keywords:** Sex addiction, Hypersexuality, Compulsive sexual behavior, Bergen–Yale Sex Addiction Scale, Personality, Psychometrics

## Abstract

Excessive problematic sexual behavior in the form of compulsive sexual behavior disorder (CSBD), hypersexuality (HS), and sex addiction has gained increasing credibility in recent years and has led to the development of various psychometric instruments to assess such behavior. However, there is still considerable controversy over the operational definition of such concepts and whether they can be used interchangeably to describe the behavior. One recently developed tool is the Bergen–Yale Sex Addiction Scale (BYSAS) based on the “components model of addiction.” The present study validated the Italian version of the BYSAS. The BYSAS was administered to a large Italian-speaking sample of Italian adults [*N* = 1230, aged 18 to 67 years] along with psychometric instruments assessing the “Big Five” personality traits, self-esteem, depression, and two other measures of addictive sexual behavior (i.e., PATHOS and Shorter PROMIS Questionnaire–Sex Subscale). Confirmatory factorial analysis supported a one-factor solution. Furthermore, the scale had good internal consistency (Cronbach’s *α* = 0.787). The BYSAS was positively associated with extroversion, openness to experience, depression, and problematic sexual behavior, and negatively associated with self-esteem, conscientiousness, emotional stability, agreeableness, and age. Based on the findings, the BYSAS is a brief, psychometrically reliable and valid measure for assessing sex addiction among Italian adults.

During the last decade, problematic excessive sexual behavior has been conceptualized in many different ways, including compulsive sexual behavior disorder (CSBD), hypersexuality, and sex addiction (among many others) (Kafka, 2010). Despite a general lack of consensus, many scholars view such excessive behavior as an impulse control disorder characterized by repetitive, intrusive, and distressing thoughts, fantasies, impulses, and sexual behaviors that negatively affect many aspects of an individual’s life (Derbyshire & Grant, 2015). Such individuals feel an obsessive pathological urge, and they try to repeatedly resist, and experience a loss of control over their own behavior (Kafka, 2010). In short, sex becomes the most important and preoccupying behavior in the individual’s life (Griffiths, 2005). More generally, excessive problematic sex has been characterized as involving sexual desires, such as fixations on partners or multiple partners, compulsive masturbation, and compulsive sex (Coleman, 1992).

CSBD and sex addiction were not included in the DSM-5 (American Psychiatric Association, 2013) for different reasons. The term “compulsive sexual behavior disorder” was not developed until 5 years later after publishing the DSM-5. Moreover, not all researchers unanimously agree on the conceptualization of “sex addiction” (e.g., Kafka, 2010). In 2018, CSBD was classified in the eleventh revision of the International Classification of Disease for Mortality and Morbidity Statistics (ICD-11), within the session of impulse control disorders and distinct from paraphilias (Kraus, et al., 2018). The estimated prevalence rate of CSBD/sex addiction is approximately 3 to 6%, although extensive representative epidemiological survey studies have not been conducted (Coleman, 1992; Carnes, 1994; Kraus et al., 2016a, b; Sussman et al., 2011).

For a diagnosis of CSBD, the ICD-11 lists symptoms of uncontrolled sexual impulses for a duration of at least 6 months with significant consequences, not linked to moral and ethical judgments, but related to the personal, familiar, social, and working sphere (Kraus, et al., 2018). CSBD manifests itself in one or more of the following behavioral patterns: (i) repetitive sexual activities that have become the central focus of the individual’s life to the point that the individual neglects health, personal care, and occupational/educational activities and responsibilities; (ii) the individual has made numerous efforts to control or significantly reduce repetitive sexual behavior but without success; (iii) the individual continues to have repetitive sexual behaviors despite the negative consequences in different areas of their life (e.g., social activities, work); and (iv) the individual continues to engage in repetitive sexual behaviors even when they derive little or no satisfaction from it (Kraus, et al., 2018). In addition, the guidelines for diagnosis were developed to help clinicians make a correct differential diagnosis, such as differentiating compulsive sexual behavior disorder from several other mental disorders and other health conditions resulting from a medical condition (e.g., bipolar disorder) (Kraus et al., 2018).

Furthermore, excessive problematic sex has been associated with several constructs and variables. For example, many studies have analyzed the relationship between internet pornography use and excessive problematic sex (e.g., Bőthe et al., 2018, 2019a, b; Chen et al., 2018; Griffiths, 2011). Moreover, excessive problematic sex has been associated with different sociocultural backgrounds, with a past of sexual abuse in childhood and adolescence being a key risk factor (Hill et al., 2007; Kraus et al., 2016a, b).

In relation to demographic variables, the literature has reported a positive relationship not only with being dependent upon sex, being young, and being male, but also with being single and having higher education (Campbell & Stein, 2015; Kafka, 2010; Karila et al., 2014; Sussman et al., 2011; Wéry & Billieux, 2017). However, some authors claim that women are underrepresented in the field of research (Dhuffar & Griffiths, 2014; Klein et al., 2014). Bőthe et al. (2018) reported a relationship between excessive problematic sex, gender, and sexual orientation. Gay men were found to be most at risk of engaging in excessive problematic sex. Furthermore, Bőthe et al., (2019a, b) examined the relationship between impulsivity, compulsivity, problematic pornography use, and excessive problematic sex. They found that impulsivity was more prominent than compulsivity in excessive problematic sex than in problematic pornography use.

Excessive problematic sex is characterized by sexual promiscuity, compulsive autoeroticism, addiction to pornographic material, and hypersexuality within a stable relationship (Bőthe et al., 2018; Kafka, 2010). Individuals engaging in excessive problematic sex feel an intense and unstoppable desire to engage in sexual activity which gradually increases in intensity in order to maintain the same satisfaction. They are unable to choose if and when to have sex, causing a significant impact on their lives with a progressive impairment of most areas of their lives (Fong et al., 2012). On a physiological level, comorbidity with other sexual dysfunctions can exist, such as premature ejaculation and delayed ejaculation (Derbyshire & Grant, 2015). The frequent uncontrolled promiscuity of such individuals is often associated with the presence of sexually transmitted diseases (McLeod & Day, 2014). These individuals usually experience anxiety, guilt, sense of inadequacy, depression, and aggressive behavior (Andreassen et al., 2018). Sexual behavior is often implemented to alleviate or avoid these withdrawal symptoms and may be explained by a dysregulation of the hypothalamic–pituitary–adrenal axis, resulting in high cortisol levels (Chatzittofis et al., 2016).

Several authors have found a correlation between depression, anxiety, and addiction (Staff, 2007) and excessive problematic sex more specifically (Lewczuk et al., 2017). Furthermore, previous studies have found that the “Big Five” personality traits (i.e., extroversion, neuroticism, agreeableness, conscientiousness, and openness) have associations with excessive problematic sex. More specifically, Shimoni et al. (2018) noted that: “individuals who are highly extroverted had sexual activity at an early age, many sexual partners, variety of sexual activity, and dangerous and careless sexual activity compared with introverted individuals. Neuroticism has been associated with liberal views about sex, unsafe sex, a problem in impulse control and negative emotions, such as anxiety, depression, and anger. Individuals with low agreeableness and conscientiousness typically enjoy unsafe sex, sexual liberalism, and impulsive risk-taking behavior compared with those with high agreeableness and conscientiousness. Finally, men with low openness tend to develop dangerous sexual behavior, such as infidelity and promiscuous sexual behavior” (Shimoni et al., 2018, p.1016). In the extant literature, specific personality traits (e.g., neuroticism and low conscientiousness) have been positively associated with many different types of addiction including sex addiction (Badii et al., 2020). Among the personality aspects that are related to sex addiction (Karila et al., 2014), high levels of extroversion and neuroticism and low levels of conscientiousness have been reported (Pinto et al., 2013; Rettenberger et al., 2016; Schmitt, 2004; Walton et al., 2017), as well as a positive association with narcissism (Kafka, 2010; Kasper et al., 2015) and the negative association with self-esteem (Badii et al., 2020; Cooper et al., 1999; Delmonico & Griffin, 2008; Doornwaard et al., 2016; Kor et al., 2014).

The growing use of technology (particularly internet use) has led to a more diversified engagement in sex such as cybersex and telephone sex (Kuss et al., 2014). Despite the high social importance and growing attention, interest in excessive problematic sex has remained at the sidelines of systematic scientific research and psychiatric classification (Kafka, 2014; Kraus et al., 2016a, b; Potenza et al., 2017).

Kafka (2010) proposed criteria for the diagnosis of “hypersexual disorder” to be included in DSM-5, highlighting how fantasies, impulses, and sexual behaviors can be due to stressful life events or dysphoric states. The individual’s consequent and vain attempt to take control causes physical or emotional damage to themselves and others, with impairment of social and professional functions. Considerable progress has been made to classify excessive problematic sex as an addiction, but there is no unanimous agreement in the scientific community.

Despite the different terms used to describe problematic excessive sexual behavior (e.g., compulsive sex, addictive sex, hypersexuality), and the lack of consensus of international researchers (e.g. Schaefer & Ahlers, 2017) on the correct terminology to be used, the present study uses the term “sex addiction” and refers to the construct outlined by Andreassen et al. (2018) who defined sex addiction “as being intensely involved with sexual activities (e.g., fantasies, masturbation, intercourse, pornography) across different media (cybersex, telephone sex, etc.). Furthermore, those with the condition report their sexual motivation is uncontrollable, and that they expend a lot of time both thinking about and being engaged in sexual activities that negatively affects many other areas in their lives” (p.2). The CSBD construct is somewhat aligned with the construct of sex addiction, although there is no specific consensus in the literature (Schaefer & Ahlers, 2017), as both refer to (i) uncontrollable sexual behaviors; (ii) failure to reduce and/or control sexual impulses; (iii) repetitive sexual activities become central to the person’s life, to the point of neglecting personal health and care or other interests, activities, and responsibilities; and (iv) sexual behavior is continuous and repetitive, despite adverse consequences that derive from it or the little or no satisfaction (World Health Organization, 2018). Arguably, the term “hypersexuality” has characteristics that overlap with the construct of “sex addiction” in that it has been defined as “dysregulated sexual behavior consisting of diminished control over sexual urges, fantasies, and behaviors, accompanied by negative consequences and significant personal distress” (Bőthe et al., 2018, p.2265).

Despite this disagreement, the hallmarks of all conceptualizations of “sex addiction” are derived from obsessive, compulsive, impulsive, and/or out of control sexual behavior (e.g., Miner et al., 2019). Moreover, Karila et al., (2014) said that: “Sexual addiction/hypersexual disorder is used as an umbrella construct to encompass various types of problematic behaviors, including excessive masturbation, cybersex, pornography use, sexual behavior with consenting adults, telephone sex, strip club visitation, and other behaviors. The adverse consequences of sexual addiction are similar to the consequences of other addictive disorders” (Karila et al., 2014).

However, at present, the sex addiction construct still remains controversial (e.g., Schaefer & Ahlers, 2017) and in many cases the terms (e.g., compulsive sexual behavior, sex addiction, hypersexuality, etc.) are used interchangeably: “Compulsive sexual behavior, also known as sex addiction, hypersexuality, excessive sexuality, or problematic sexual behavior, is characterized by repetitive and intense preoccupations with sexual fantasies, urges, and behaviors that are distressing to the individual and/or result in psychosocial impairment” (Derbyshire & Grant, 2015, p.37).

Although the terms are often used interchangeably, according to different authors, compulsive sex, sex addiction, and hypersexuality are different constructs. As Andreassen et al. (2018) note: “There has been much debate over many years as to whether this behavior is best conceptualized as an obsessive–compulsive disorder, an addiction, or a disorder of impulse-control, and consequently been explained according to different conceptual models…In line with this, a limitation of prior research is the absence of a general consensus about how sex addiction should be determined, understood, and assessed” (p.2).

Due to the lack of consensus over its conceptualization, psychometric scales used in the past decade to assess sex addiction differ in procedure, development, factorial structure cutoffs, and psychometric properties (Campbell & Stein, 2015; Hook et al., 2010; Karila et al., 2014; Wéry & Billieux, 2017). Such a rapid development of many assessment tools has also led to various methodological weaknesses. For instance, many scales have either been utilized in small clinical samples that are not representative of the population (Karila et al., 2014a, b). Furthermore, some of these scales are very specific for a particular type of population (females, males, heterosexual, homosexual, etc.) (Carnes, 1991; Carnes & Weiss, 2002; Carnes & O’Hara, 2000), or the medium (e.g., online sexual behavior) (Carnes et al., 2010; Wéry & Billieux, 2016). The contemporary study of sex addiction has led to the development of increasingly appropriate tests such as the short Internet Addiction Test (Young, 1998) being adapted to assess online sexual activities (e.g., IAT-Sex; Laier et al., 2013; Pawlikowski et al., 2013; Wéry et al., 2016), Screening Test for Sexual Addiction (SAST; Carnes, 1989), the SAST-Revised (SAST-R; Carnes et al., 2010), Shorter PROMIS Questionnaire (SPQ-S; Christo et al., 2003), and PATHOS sex addiction screen (Carnes et al., 2012). In addition, there are validated scales that evaluate and conceptualize hypersexuality as a compulsive, impulsive, and/or sexual dysregulation disorder (e.g. Kalichman & Rompa, 1995; Reid et al., 2011).

Many previous scales assessing various behavioral addictions have been based on the six criteria in the components model of addiction (Griffiths, 2005). Applied to sex, the six criteria are *salience*, over-preoccupation with sex or wanting sex; *mood modification*, using sex to modify mood state; *tolerance*, increasing the amounts of sex over time; *withdrawal*, unpleasant emotional and physical symptoms when unable to have sex; *conflict*, compromising all areas of life as a result of sex (e.g., relationships, occupation/education, social activities, etc.); and *relapse*, returning to previous problematic patterns of sexual behavior after a period of abstinence or control.

Another limitation of these instruments is the response structure. The SAST-R (Carnes et al., 2010) and PATHOS (Carnes et al., 2012) both use dichotomous (yes/no) responses that are not expansive enough in assessing complex problem and should ideally be assessed using a continuous response dimension (e.g., Likert scale) (Walters et al., 2011; Carvalho et al., 2015). Finally, there is the issue of brevity. In a systematic review of 24 self-report hypersexuality scales, Womack et al. (2013) reported that the scales assessed had an average of 32.5 items. Scale brevity is a key criterion for increasing scale efficacy, particularly in the context of research (Koronczai et al., 2011).

Some of the core components of behavioral addiction are not highlighted in the most common sex addiction scales (Griffiths, 2005). The growing interest in sex addiction research has been accompanied with a rapid development of many other instruments, focusing on brevity, and psychometric validation (Griffiths, 2012; Paz et al., 2019). Focusing on brevity and efficacy is the Bergen–Yale Sex Addiction Scale (BYSAS) (Andreassen et al., 2018). At present, there is no scale that assesses sex addiction in the Italian territory which includes the core components of behavioral addiction (Griffiths, 2005) and that is (i) short (i.e., having few items), (ii) psychometrically robust, and (iii) has items that are assessed on a Likert scale. More specifically, the scale is based on the six core components of behavioral addiction comprising salience, mood modification, tolerance, withdrawal symptoms, conflicts, and relapse/loss of control. Consequently, the aim of the present study was to translate and validate the BYSAS test in the Italian context to have a new research tool that has the following characteristics: (i) be short with only a few items, (ii) based on core addiction criteria, and (iii) be psychometrically valid and reliable. As well as evaluating the scale’s psychometric properties, it was hypothesized that the BYSAS would to be positively correlated with theoretically related constructs and variables (being male, single, higher educated, extroversion, and openness) and negatively correlated with theoretically divergent constructs and variables (age, agreeableness and conscientiousness) (Andreassen et al., 2018; Nunnally & Bernstein, 1994).

## Methods

### Participants and Procedure

Participants were recruited by posting links to the survey in different Italian online forums and social media communities (e.g., *Facebook*), via a link that advertised a survey to be completed on the *Google Forms* platform. The link was distributed by the research team, inviting individuals to participate voluntarily, anonymously, and without any reward. During a 20-day period (from July 1, 2020, to July 19, 2020), 1,230 voluntary participants responded to the online survey, which took around 15–20 min to complete. The inclusion criteria were that participants had to be (i) at least 18 years old and (ii) Italian-speaking citizens. All the participants completed the survey anonymously after providing their informed consent. All participants completed all parts of the survey, so there were no missing data.

### Measures

#### Sociodemographics, Life Habits, and General Questions Related to Sexual Behavior

The survey included questions concerning the sociodemographic aspects of the participants (e.g., sex, age, educational level, relationship status, work, sexual orientation). In addition, questions were asked about the participants’ sexual behavior (on a five-point scale from ‘never’ to ‘very often’) including weekly masturbation activity, weekly sexual activity, whether they accessed online pornography websites (yes/no), how often they accessed online pornography, how often they masturbated using online pornography, and rating on the quality of their sex life (‘very low’ to ‘very high’). Participants were also asked the number of sexual contacts in the past 6 months. If there was at least one sexual contact within the past 6 months, participants were asked to indicate how satisfied they were with the frequency and the quality of them (‘not satisfied’ to ‘very satisfied’). Participants were also asked whether they (i) had ever experienced sexual abuse (yes/no), (ii) had unprotected sex (‘never’ to ‘very often’), and (iii) ever engaged in group sex (‘never’ to ‘very often’). Another question asked was: “During the lockdown due to Covid-19, did your masturbation sexual activity increase?” rated on a scale of 1 (*not at all*) to 5 (*very much*). Other variables examined were weekly sleep quality of the participants (very poor to very good), loneliness (‘never’ to ‘most of the time’), and alcohol and drug use (never to very often).

#### Rosenberg’s Self-Esteem Scale (RSES; Rosenberg, 1965)

The 10-item Italian version of RSES (Prezza et al., 1997) was used to assess self-esteem (e.g., “On the whole, I am satisfied with myself”) using a four-point Likert type scale from 0 (*strongly disagree*) to 3 (*strongly agree*). Scores range between 0 and 30 and higher scores indicate greater self-esteem. Cronbach’s alpha in the present study was excellent (*α* = 0.90).

#### Ten-Item Personality Inventory (TIPI; Gosling et al., 2003)

The 10-item Italian version of the TIPI (Chiorri et al., 2015) was used to assess the Big Five personality traits (i.e., openness, conscientiousness, extraversion, agreeableness, and neuroticism [emotional stability]). The items are assessed on a seven-point scale from 1 (*strongly disagree*) to 7 (*strongly agree*). Example items include “I see myself as extraverted” and “I see myself as open to new experiences.” The scores range from 2 to 14 on each trait, and higher scores indicate a greater propensity for the given personality trait. Cronbach’s alpha in the present study was low (*α* = 0.50) but the original authors claimed that even a low *α* value still makes the scale usable (Gosling et al., 2003).

#### Shorter PROMIS Questionnaire–Sex Subscale (SPQ-SS; Christo et al., 2003; Lefever, 1988)

The complete SPQ-SS originally comprised 16 scales. In the present study, only the 10-item *sex* subscale of the SPQ was used. Each item (e.g., “I would take an opportunity to have sex despite having just had it with somebody else”) is assessed on a six-point scale from 0 (completely disagree) to 5 (completely agree). Score can range from 0 to 50, with higher scores indicating greater sex addiction. The scale was translated from English into Italian in the present study following the protocol described by Beaton et al. (2000). More specifically, the items were independently translated by a mother tongue translator and utilized internationally accepted practices for scale translation. The scale has not been validated previously in Italy, although previous Italian studies have used it (e.g., Pallanti et al., 2006). The present study used the scale to assess convergent and criterion validity, replicating the original BYSAS validation study (Andreassen et al., 2018). Cronbach’s alpha in the present study was good (*α* = 0.72).

#### Adult PROMIS Emotional Distress/Depression-Short Form (APEDD-SF; Cella et al., 2010)

The eight-item Italian version of the APEDD-SF (Fossati et al., 2015) was used to assess depression among individuals aged 18 years and older. The items (e.g., “I feel useless”) are answered by a scale of 1 (*never*) to 5 (*very frequently*) with scores ranging from 8 to 40, with a higher score indicating a higher level of depression. Cronbach’s alpha in the present study was excellent (*α* = 0.947).

#### PATHOS (Carnes et al., 2012)

The six-item PATHOS scale is a screening instrument that assesses sexual addiction utilizing dichotomous yes/no answers. Example items include “Do you often find yourself preoccupied with sexual thoughts?” and “Has anyone been hurt emotionally because of your sexual behavior?” If participants answer positively to four or more items, there is a likelihood of sexual addiction. The scale was translated from English into Italian in the present study following the protocol described by Beaton et al. (2000) and briefly described above. In the present study, although the Cronbach’s alpha was low (*α* = 0.512), the Kuder-Richardson-21 reliability coefficient (K-21), more suitable when all scale variables are dichotomous (Kuder & Richardson, 1937]), was sufficient (0.67). As indicated by the original developers, the PATHOS is suitable for an initial screening. However, the scale was considered reliable for the purposes of the present study.

#### Bergen–Yale Sex Addiction Scale (BYSAS; Andreassen et al., 2018)

The six-item BYSAS assesses the risk of sex addiction and is responded to on a five-point scale from 0 (*very rarely*) to 4 (*very often*) based on the components model of addiction (Griffiths, 2005). Applied to sex, the six criteria are *salience*, over-preoccupation with sex or wanting sex; *mood modification*, using sex to modify mood state; *tolerance*, increasing the amounts of sex over time to maintain high levels of satisfaction; *withdrawal*, unpleasant emotional and physical symptoms when unable to have sex; *conflict*, compromising all areas of life as a result of sex (e.g., relationships, occupation/education, social activities, etc.); and *relapse*, returning to previous problematic patterns of sexual behavior after a period of abstinence or control. BYSAS scores range from 0 to 24 with a higher score indicating a greater risk of sex addiction. BYSAS is a generic sex addiction screening tool, as it does not focus on particular demographic groups (e.g. male or female, gay or heterosexual) or a particular medium (e.g. online sex). The scale was translated from English into Italian in the present study following the protocol described by Beaton et al. (2000) and described briefly above. Example of items are “How often during the past year have you spent a lot of time thinking about sex/masturbation or planned sex?” and “How often during the past year have you tried to cut down on sex/masturbation without success?” Cronbach’s alpha in the present study was good (*α* = 0.787).

#### Ethics

The study was approved by the ethics committee of the Italian Group Cognitive Behavioral Psychotherapy Association. Informed consent was obtained from all participants and they all participated voluntarily.

#### Statistical Analysis

Before analyzing the data obtained from the sample, univariate normality of the data was verified using the guidelines proposed by Muthén and Kaplan (1985) (i.e., skewness and kurtosis in the − 1 to + 1 range). Descriptive statistics concerning the items (i.e., frequencies, percentages) were calculated. The statistical analyses carried out were as follows: (i) descriptive statistics of the BYSAS items (i.e., means and standard deviations of the main items); (ii) construct and criterion validity of the Italian BYSAS; (iii) the reliability of the scale, examined via composite reliability (CR) (values of CR greater than 0.7 are associated with strong reliability of the test; Fornell & Larcker, 1981). The evaluation of the factor structure and the dimensionality of the Italian BYSAS was assessed utilizing confirmatory factor analysis (CFA). Specific indices were also calculated to ascertain the unidimensionality of the test which met the following criteria (Ferrando & Lorenzo-Seva, 2013): UNIQUE (one-dimensional congruence > 0.95), ECV (explained common variance > 0.80), and MIREAL (average of absolute loads residues of the object < 0.30). The extraction of the factors for CFA was carried out with the Diagonal Weighted Least Squares estimation (DWLS) method.

With regard to the CFA, the indices recommended by Hair et al. (2019) were adopted as follows: NNFI (non-normed fit index ≥ 0.95), CFI (comparative fit index ≥ 0.95), GFI (goodness of fit index ≥ 0.95), AGFI (adjusted goodness of fit index ≥ 0.95), RMSEA (root mean square error of approximation ≤ 0.08), and RMSR (root mean square of residuals ≤ 0.8). The reliability of the data was assessed through the following indices: Cronbach’s alpha (*α*) (Cronbach, 1951), McDonald’s omega (*ω*) (McDonald, 1999), and composite reliability (CR). The replicability of the construct and the quality of the factorial solution found were evaluated with the use of the H-coefficient (Hancock & Mueller, 2001; Loevinger, 1948; Mokken, 1971) with a cutoff of 0.50. The analyses were carried out using FACTOR v.10.10.3 (Lorenzo-Seva & Ferrando, 2006), SPSS Statistics v.25 (IBM Corporation, 2011), Amos v.23 (Arbuckle, 2013a, b), JASP version 0.13.1 (JASP Team, 2020), Mann–Whitney U Test Calculator (2017), and the R package lavaan (Yves Rosseel, 2012).

## Results

### Sociodemographic Characteristics

Although the main sociodemographic characteristics (summarized in Table 1) of the sample were not balanced (26.7% males, 73.1% females, other 0.2%; mean age 24.9 years [SD ± 5.60]; education level: 65.5% university-level degree, 34.1% high school degree, and 0.3% lower secondary degree), the high number of participants ensured robust data analysis. Other characteristics in the sample included 67.3% students, 25.9% employed, and 6.7% unemployed; 58.5% were in a non-married relationship, 37.2% were single, 3.4% were married, 0.5% were divorced, 0.2% were separated, and 0.2% widower. As for sexual orientation, 82.1% were heterosexual, 9.6% were bisexual, 5.5% were homosexual, and 2.8% were for others.

**Table 1:** Main descriptive statistics of the sample (*N* = 1230)

		Frequency	%
Gender	Other	3	0.2
	Female	899	73.1
	Male	328	26.7
Educational level	Secondary school	4	0.3
	High school	419	34.1
	University	807	65.6
Work condition	Unemployed	82	6.7
	Worker	319	25.9
	Retired	1	0.1
	Student	828	67.3
Relationship status	Divorced	6	0.5
	Fiancé	720	58.5
	Separate	3	0.2
	Single	457	37.2
	Married	42	3.4
	Widower	2	0.2
Sexual orientation	Other	34	2.8
	Bisexual	118	9.6
	Heterosexual	1010	82.1
	Homosexual	68	5.5

During the lockdown due to COVID-19, 7.3% of participants greatly increased their sexual or masturbation activity, with an mean average of 2.32 (out of 5) (SD = 1.29) and 6.2% greatly increased their access activity to websites related to pornography, with a mean of 1.77 (out of 5) (SD = 1.29). In addition, 2.6% of the participants used alcohol or drugs very often, and 7.4% of the participants had experienced sexual abuse. The weekly hours spent by participants watching sexual content online were 1.77 h (SD = 2.70). In relation to the psychometric tests, the distribution of the mean scores was as follows: Italian BYSAS = 7.61 out of 24 (SD = 4.80), PATHOS = 1.05 out of 6 (SD = 1.15), Adult PROMIS Emotional Distress/Depression-Short Form = 19.84 out of 40 (SD = 9.33), Shorter PROMIS Questionnaire–Sex Subscale = 15.06 out of 50 (SD = 6.59), Rosenberg Self-Esteem Scale = 18.30 out of 30 (SD = 7.31) (see Table 2). The results of the other main questions asked to the participants are summarized in Tables 2 and 3.

**Table 2:** Descriptive statistics of the main tests used (*N* = 1230)

	*N*	Mean	Standard deviation	Skewness		Kurtosis	
	Statistic	Statistic	Statistic	Statistic	Standard error	Statistic	Standard error
BYSAS*****	1230	7.61	4.80	0.69	0.07	0.31	0.14
Depression**	1230	19.84	9.33	0.59	0.07	− 0.82	0.14
Sex subscale***	1230	15.06	6.59	2.18	0.07	5.87	0.14
Self-esteem****	1230	18.30	7.31	− 0.33	0.07	− 0.87	0.14
PATHOS	1230	1.05	1.15	1.50	0.07	3.12	0.14
Extroversion*	1230	5.72	2.43	0.04	0.07	− 0.96	0.14
Agreeableness*	1230	7.25	1.94	− 0.25	0.07	− 0.76	0.14
Conscientiousness*	1230	7.99	1.78	− 0.63	0.07	0.01	0.14
Emotional stability*	1230	5.33	2.31	0.32	0.07	− 0.79	0.14
Openness to* experiences	1230	7.01	1.83	− 0.24	0.07	− 0.31	0.14

Note: *Dimensions of the Ten-Item Personality Inventory **Adult PROMIS Emotional Distress/Depression-Short ***Shorter PROMIS Questionnaire–Sex Subscale **** Rosenberg Self-Esteem Scale ***** Bergen–Yale Sex Addiction Scale

**Table 3:** Main questions addressed to the participants

		Life quality	Visit online sex sites	Frequency of masturbation (in a week)	Sex frequency (in a week)	Quality of sleep	Unprotected sex frequency	Group sex frequency	Perceived loneliness
N		1230	1230	1230	1230	1230	1230	1230	1230
Mean		2.94	2.75	2.74	2.36	3.22	2.31	1.11	2.49
SD		1.43	1.34	1.29	1.18	1.17	1.58	0.43	1.25
Minimum		1.00	1.00	1.00	1.00	1.00	1.00	1.00	1.00
Maximum		5.00	5.00	5.00	5.00	5.00	5.00	5.00	5.00

Note = All questions assessed on a scale from 1 (never) to 5 (very often). *SD* standard deviation

### Confirmatory Factor Analysis (CFA) of the Italian Bergen–Yale Sex Addiction

**Fig. 1:**
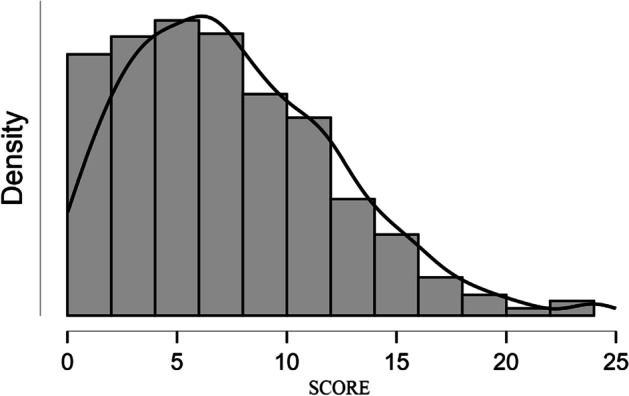
BYSAS total score distribution

The present study analyzed the distribution of the six BYSAS items (Fig. 1). Most items (see Table 4) were distributed asymmetrically (i.e., a positive asymmetric distribution), with the highest frequencies in the lowest values. As for asymmetry and kurtosis, most of the items were distributed in a non-normal way (the items do not fall within the range of ± 1; see Muthén & Kaplan, 1985). To investigate and analyze the factorial structure, since there is no unequivocal consensus in the literature (see Bollen & Long, 1993; Boomsma, 2000), different goodness of fit (GOF) adaptation indices were used to confirm the dimensionality of the BYSAS. In this specific case, since the items (see Table 3) were distributed in a non-normal way, parallel analysis (PA)/Diagonal Weighted Least Squares estimation method (DWLS, bias-corrected and accelerated [Bca] with 95% confidence interval, 1000 random sample) was used for confirmatory factorial analysis (ten Berge & Kiers, 1991; Baglin, 2014; Mindrila, 2010; Krijnen, 1996).

**Table 4:** Italian Bergen–Yale Sex Addiction Scale (BYSAS) item distribution

	*N*	Mean	Standard deviation	Skewness		Kurtosis	
	Statistic	Statistic	Statistic	Standard error	Statistic	Standard error	Statistic
Item 1	1230	1.95	1.16	0.07	0.00	0.13	− 0.83
Item 2	1230	2.11	1.18	0.07	− 0.20	0.13	− 0.84
Item 3	1230	1.50	1.38	0.07	0.38	0.13	− 1.19
Item 4	1230	0.62	1.03	0.07	1.68	0.13	1.97
Item 5	1230	0.93	1.18	0.07	1.08	0.13	0.09
Item 6	1230	0.46	0.87	0.07	2.06	0.13	3.87

### Confirmatory Factor Analysis

Since there is no univocal academic consensus about the indicators to evaluate a model reliability (Bollen & Long, 1993; Boomsma, 2000; Hoyle & Panter, 1995), goodness of fit was evaluated using several indicators, as in the EFA. The results of the confirmatory analysis are as follows: *χ*^2^ = 14.99 (df = 8, *n* = 1230), *p* = 0.06 (i.e., not significant at *p* < 0.05: chi-square is very sensitive to the size of the sample, so several indices were used; Kline, 2016), TLI = 0.99, CFI = 1, RMSEA = 0.03, and SRMR = 0.03 (< 0.09; Hu & Bentler, 1999). Furthermore, all items were positively related to each other (min = 0.18, max = 0.81). Furthermore, most factor loadings were high on all items, with only item 6 loading under 0.5 value (min = 0.40, max = 0.89, i.e., λij ≥ 0.50; Ferguson & Cox, 1993). However, all items had a statistically significant factor loading (*p* < 0.01) and residual covariance between item 1 (preoccupation) and item 2 (tolerance), in a similar way to the original research (Andreassen et al., 2018).

These results demonstrate that the BFAS presented an excellent fit to the data (see Tables 5, 6, and 7 for details). In addition, to investigate the general stability, replicability, and cross-validation stability of the factor, the following indices were obtained: coefficient of fidelity (O-COF) = 0.808, fidelity index (FI) = 0.926 (for an O-COF or a FI, value equal or larger than 0.90 would suggest an acceptable measurement accuracy; Ferrando & Lorenzo-Seva, 2019).

**Table 5:** Factor loadings of the BYSAS (*N* = 1,230)

										95% confidence interval		
Factor	Indicator	Symbol	Estimate		Std. error			*Z* value	*p*	Lower		Upper
	BYSAS_01	λ1		0.69		0.03	21.01		< 0.001	0.62	0.75	
	BYSAS_02	λ2		0.77		0.03	23.18		< 0.001	0.70	0.83	
	BYSAS_03	λ3		0.89		0.03	25.85		< 0.001	0.83	0.96	
	BYSAS_04	λ4		0.57		0.03	21.10		< 0.001	0.52	0.62	
	BYSAS_05	λ5		0.81		0.03	24.92		< 0.001	0.74	0.87	
	BYSAS_06	λ6		0.40		0.02	17.29		< 0.001	0.36	0.45

**Table 6:** Fit indices of the BYSAS (*N* = 1,230)

Index	Value
Comparative fit index (CFI)	1.00
Tucker-Lewis index (TLI)	0.99
Bentler-Bonett non-normed fit index (NNFI)	0.99
Bentler-Bonett normed fit index (NFI)	0.99
Parsimony normed fit index (PNFI)	0.53
Bollen’s relative fit index (RFI)	0.99
Bollen’s incremental fit index (IFI)	1.00
Relative noncentrality index (RNI)	1.00

**Table 7:** Other fit measures of the BYSAS (*N* = 1,230)

Metric	Value
Root mean square error of approximation (RMSEA)	0.03
RMSEA 90% CI lower bound	0.00
RMSEA 90% CI upper bound	0.05
RMSEA *p* value	0.97
Standardized root mean square residual (SRMR)	0.03
Hoelter’s critical *N* (*α* = 0.05)	1269.10
Hoelter’s critical *N* (*α* = 0.01)	1643.87
Goodness of fit index (GFI)	1.00
McDonald fit index (MFI)	1.00
Expected cross-validation index (ECVI)	0.03

After the CFA, different indexes of reliability (i.e., internal consistency) and validity (i.e., discriminant and convergent validity) were investigated. To analyze the reliability of the BYSAS and internal consistency, Cronbach’s alpha (see Table 8), factor determinacy index (FDI), McDonald’s omega, H-coefficient, and composite reliability were calculated (Raykov, 1997). Cronbach’s alpha in the present study was 0.787 and could not be improved by removing any items. The FDI was 0.929, EAP marginal reliability was 0.864, expected percentage of true differences (EPTD) was 90.9% (EPTDs above 90% and marginal reliabilities above 0.80 are recommended for a good model; Ferrando & Lorenzo-Seva, 2018), McDonald’s omega was 0.787 and Gutmann’s λ6 was 0.779 (λ6 > 0.70 is sufficient to be considered reliable [Gallardo-Vázquez & Folgado-Fernández, 2020], see Table 7 and Fig. 3 for details), H-coefficient was 0.508 (*H* > 0.5, is considered strong; Sijtsma & Molenaar, 2002), Generalized H Index (G-HI) was 0.864 (G-HI values > 0.80 suggest a well-defined latent variable; Hancock & Mueller, 2000), and the CR was 0.806 (for a narrowly defined construct with five to eight items to meet a minimum threshold of 0.80; Netemeyer et al., 2003).

**Table 8:** Item reliability statistics of the BYSAS

	If item dropped
Item	Cronbach’s α*
BYSAS_01	0.738
BYSAS_02	0.725
BYSAS_03	0.760
BYSAS_04	0.768
BYSAS_05	0.747
BYSAS_06	0.782

*Point estimate *α* = 0.787

Subsequently, the correlation matrix between BYSAS and the other divergent/concurrent constructs considered in the study were calculated in order to investigate if the observed correlation path confirmed the construct validity of the BYSAS. Direction and strength of the coefficients were assessed following Cohen’s (1988) interpretation (see Table 9) and were consistent with the theoretically predicted relationship.

**Table 9:** Pearson’s correlation matrix (*N* = 1,230)

Variable	BYSAS		SPQ-SS		RSES		APEDD-SF	PATHOS
1. BYSAS	—							
2. SPQ-SS		0.5328 ***	—					
3. RSES		− 0.1843 ***	− 0.03290	—				
4. APEDD-SF		0.2614 ***	0.1301 ***	− 0.7253 ***		—		
5. PATHOS		0.4644 ***	0.4364 ***	− 0.1535 ***		0.1880 ***		—

Note: *p < 0.05, **p < 0.01, ***p < 0.001. *BYSAS* Bergen–Yale Sex Addiction Scale; *APEDD-SF* Adult PROMIS Emotional Distress/Depression-Short Form; *SPQ-SS* Shorter PROMIS Questionnaire–Sex Subscale; *RSES* Rosenberg’s Self-Esteem Scale

The statistically significant positive correlations between the BYSAS and the SPQ-SS (intermediate), APEDD-SF (small), and PATHOS (intermediate) support the scale’s convergent validity. Conversely, the statistically significant negative correlation between the BYSAS and the RSES (small) supports the evidence of divergent validity (see Table 9). The Italian BYSAS (total score) correlated positively with loneliness (*r* = 0.292 *p* < 0.01), frequency of use of visiting internet sex sites (*r* = 0.395 *p* < 0.01), weekly masturbation frequency (*r* = 0.434 *p* < 0.01), weekly sexual intercourse frequency (*r* = 0.063 *p* < 0.05), number of sexual encounters in the past 6 months (*r* = 0.049, *p* < 0.05), frequency of masturbation viewing pornographic material on the internet (*r* = 0.363 *p* < 0.01), hours spent viewing images/videos on the Internet (*r* = 0.258 *p* < 0.01), depression (*r* = 0.258, *p* < 0.01), and gender (*r* =  − 0.25 *p* < 0.01). BYSAS correlated negatively with age (*r* =  − 0.055 *p* < 0.05), the quality of one’s sexual life referred to the last month (*r* =  − 0.061 *p* < 0.05), the quality of sleep referred to the last week (*r* =  − 0.178, *p* < 0.01), self-esteem (*r* =  − 0.182, *p* < 0.01), agreeableness (*r* =  − 0.098, *p* < 0.01), conscientiousness (*r* =  − 0.265, *p* < 0.01), emotional stability (*r* =  − 0.105, *p* < 0.01), and education (*r* =  − 0.037, *p* < 0.05). There were no significant findings in relation to extraversion and openness to experience. Furthermore, a two-way ANOVA was conducted to investigate the relevance of two between-participant factors (gender and relationship status) on the BYSAS score and in particular the interaction effect of these independent variables, and the results of the model are presented in Table 10.

**Table 10:** Gender-BYSAS total score and relationship status

Cases	Sum of squares	df	Mean square	F	p	η2	η2 p	ω2
Gender	1533.976	1	1533.976	71.190	< 0.001	0.055	0.055	0.054
Relationship status	136.928	1	136.928	6.355	0.012	0.005	0.005	0.004
Gender × relationship status	4.593	1	4.593	0.213	0.644	1.639e -4	1.743e -4	0.000
Residuals	26,352.744	1223	21.548					

**Fig. 2:**
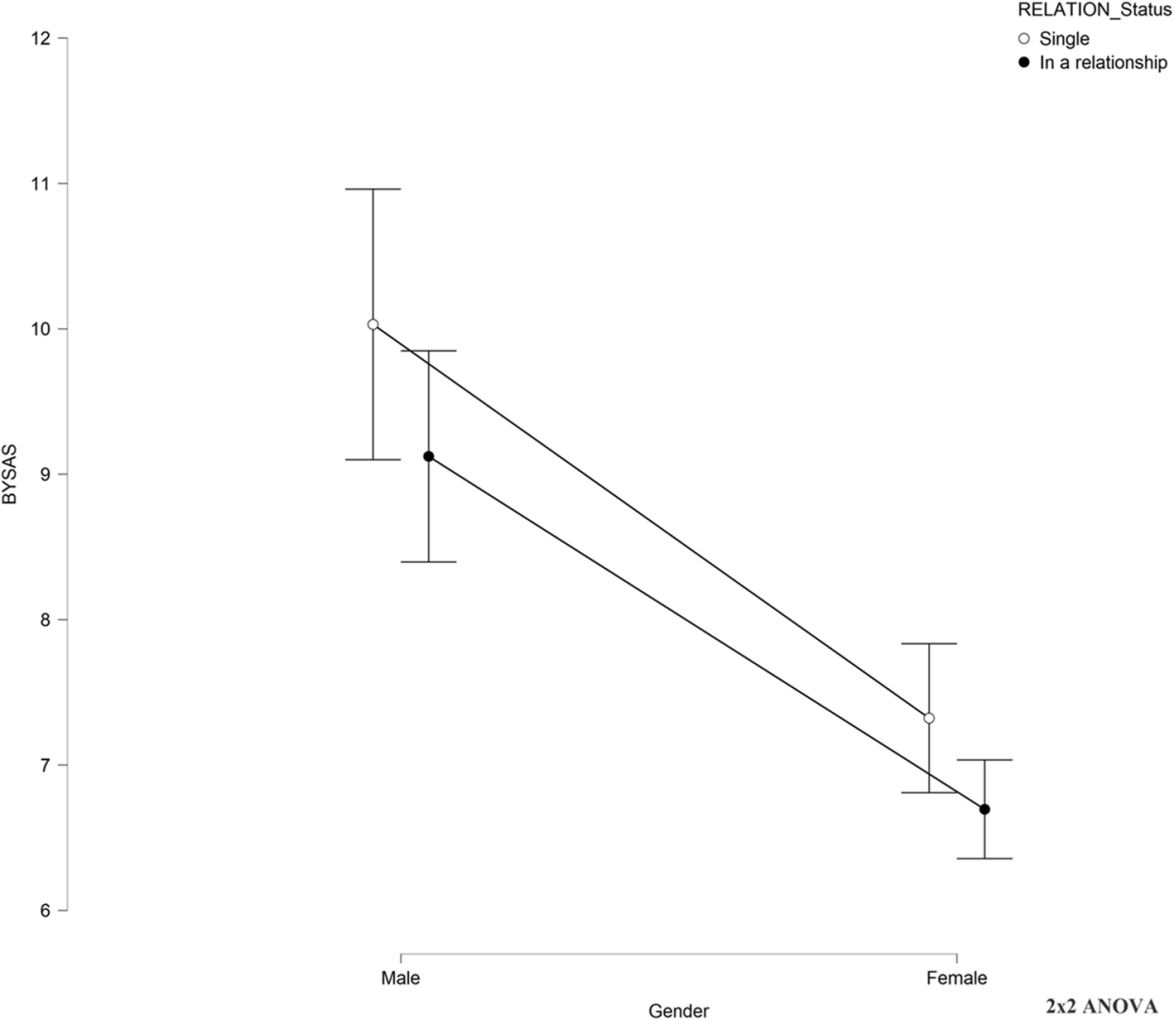
2 × 2 ANOVA

Initial results of the 2 × 2 ANOVA showed that the interaction effect between gender and relationship status on BYSAS was not statistically significant (*F* [1, 1223] = 0.213, *p* = 0.644). Therefore, an analysis of the main effects was performed, which indicated the main effects were statistically significant for both gender (*F* [1, 1223] = 71.190, *p* < 0.001, partial *η*^2^ = 0.055) and relationship status (*F* (1, 1223) = 6.355, *p* < 0.05, partial *η*^2^ = 0.005). All pairwise comparisons were run where reported 95% confidence intervals and *p* values are Bonferroni-adjusted (see Table 10 and Fig. 2). Males had significantly higher scores on the BYSAS than female (Z =  − 7.735, *p* < 0.01).

**Fig. 3:**
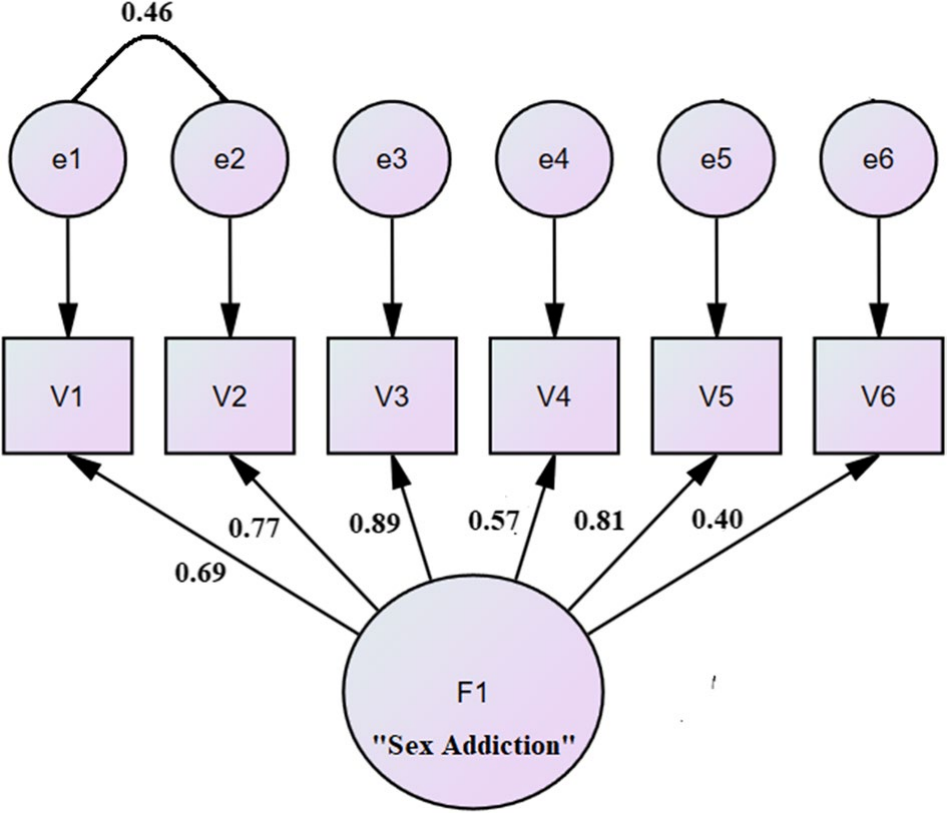
Bergen–Yale Sex Addiction Scale CFA model

Through a multiple linear regression, a model was built comprising the following independent variables: age, depression, gender, self-esteem, extroversion, agreeableness, conscientiousness, emotional stability, openness to experiences, education level, with the dependent variable of the BYSAS total score. The results showed *R* = 0.419, *R*^*2*^ = 0.175, *F* = 25.9 (df = 10), and *p* < 0.001. The model was significant and explained 17.5% of the variance of the BYSAS test total score (see Table 11 and Fig. 3).

**Table 11:** Multiple regression of total score of the BYSAS (*N* = 1,230)

	Non-standardized coefficients		Standardized coefficients	*t*	Sign. *
	*β*	Standard error	*β*		
(Constant)	5.28	1.45		3.63	0.00
Age*	− 0.04	0.02	− 0.05	− 1.93	**0.04**
Depression*	0.14	0.02	0.27	6.91	**0.00**
Gender*	2.63	0.29	0.25	9.09	**0.00**
Self-esteem	0.03	0.03	0.05	1.19	0.24
Extroversion	0.08	0.06	0.04	1.32	0.19
Agreeableness	− 0.06	0.07	− 0.02	− 0.83	0.41
Conscientiousness*	− 0.50	0.08	− 0.19	− 6.64	**0.00**
Emotional Stability	− 0.09	0.07	− 0.04	− 1.41	0.16
Openness to Experiences	0.09	0.07	0.03	1.15	0.25
Education Level	0.24	0.26	0.02	0.91	0.36

^*^Significant for *p* < 0.05, Note: *β* beta, linear regression coefficient

## Discussion

The present study investigated the psychometric properties of the Italian version of Bergen–Yale Sex Addiction Scale (BYSAS) (Appendix 1). The results indicated a stable unidimensional structure confirming the findings of the original validation study by Andreassen et al. (2018). The analysis of the Italian BYSAS reliability and validity coefficients showed sound and consistent psychometric properties of the scale. All the hypotheses were confirmed. As shown in previous research (e.g., Andreassen et al., 2018), sex addiction is often comorbid with depression and low self-esteem; therefore, the construct validity of the Italian BYSAS was strengthened by its positive and statistically significant correlations with the APEDD-SF (which assesses the severity of depression) and by its negative and statistically significant correlation with the RSES (which assesses self-esteem). Furthermore, the significant correlation between the BYSAS and the two other scales that assess sex addiction issues used in the present study (i.e., the PATHOS and PROMIS Sex Subscale) strongly supports not only its concurrent validity (which is determined by observing how much the instrument correlates with other valid instruments in assessing the same characteristic), but also its usefulness and efficacy as a short-form scale. The results of reliability and validity of the scale reflect what was found in other versions of the scale such as its Hebrew version (Paz et al., 2019).

The results of the correlation analysis also confirmed the association between sex addiction and co-occurring problematic behaviors related in the sexual sphere as well as a negative correlation between BYSAS score and age (i.e., the higher the age, the lower the sex addiction score). This result is in line with previous studies: “some types of excessive sex can be physically demanding and that sexual libido tends to decrease as individuals get older, it is perhaps unsurprising that sex addiction is associated with younger age” (Andreassen et al., 2018, p.10). Additionally, participants who were both single and male had higher scores on the BYSAS. This could reflect the fact that “the majority of individuals seeking professional help for addictive sex behavior are men and they also reflect that women to a lesser extent come forward due to potentially more social stigma and inner shame than men” (Andreassen et al., 2018 p.10). Furthermore, according to previous literature, single people are more motivated to satisfy their sexual desires, compared to those who are in a more stable relationship (Kafka, 2010, Campbell & Stein, 2015; Ballester-Arnal et al., 2013; Sun et al., 2014). Regarding personality aspects, BYSAS total score was positively correlated with extraversion, and openness to experience, and negatively associated with agreeableness, conscientiousness, and emotional stability. Some personality traits (e.g., low conscientiousness, neuroticism) have consistently been found as predictors of addiction (Shimoni et al., 2018).

Similarly, Andreassen et al. (2018) found that self-esteem was inversely related to the sex addiction items. Finally, in relation to the quality of sleep, the results were consistent with previous studies (e.g., Brunborg et al., 2011). More specifically, the total BYSAS score was negatively associated with the perceived quality of sleep. This is in line with previous studies that have shown that the presence of different forms of addiction (e.g., internet, addiction, social media addiction) are related to decreased sleep quality (Alimoradi et al., 2019).

The present study presents some limitations that need to be addressed. More specifically, the cross-sectional research design, in addition to the convenience sample and self-reported data, can result in biased results (e.g. content sensitive response bias such as social desirability). Indeed, although the survey was anonymous, participants may have been ashamed to report their problematic sexual behaviors (Andreassen et al., 2018). Regarding the non-random and voluntary sample, although relatively large scale, it cannot be considered representative of the entire population (and therefore generalizability of the findings is limited) and the sample included a higher proportion of females (probably due to advertisement on online groups related to the psychology faculty). It should also be noted that the PATHOS had a low Cronbach’s alpha but the SPQ-SS had good reliability and there were no reliability issues with the latter scale. As indicated by the original developers, the PATHOS is suitable for an initial screening. A further limitation of this study is the lack of measurement invariance by gender and age. This was not possible to do, given the imbalance of the sample in relation to these variables. Future research, with a more representative sample, should address the issue of measurement invariance.

Based on the psychometric analysis conducted in the present study, the BYSAS is a short, reliable, and valid scale to assess sex addiction when a brief screening is necessary. The analysis demonstrated that the BYSAS yielded strong psychometric properties in terms of factor structure and reliability. However, the application of the findings to the general population should be made with caution due to fundamental differences in men’s and women’s sexual activity (e.g., male use of pornography is much higher than females) (Bőthe, et al., 2019a, b). In the future, researchers should further investigate construct validity concerning clinical samples to understand if it is possible to establish the pathological screening efficacy of the scale, identifying for example optimal cutoff that could reliably distinguish between pathological and non-pathological cases. Finally, it should also be noted that the Italian BYSAS demonstrated excellent model fit and adequate reliability. This makes it a valuable tool for assessing the severity of sex addiction among Italian adults, especially in the context of epidemiological research.

## Appendix

### Appendix 1 Italian Version of Bergen–Yale Sex Addiction Scale

1. Ti è capitato di trascorrere molto tempo pensando al sesso/masturbazione o a pianificare i tuoi incontri sessuali?
2. Ti è capitato di provare un forte desiderio di masturbarti/fare sesso sempre di più?
3. Ti è capitato di usare il sesso/masturbazione per dimenticare/fuggire dai tuoi problemi personali?
4. Hai provato a ridurre il tempo trascorso a masturbarti/fare sesso senza riuscirci?
5. Ti è capitato di diventare triste/irrequieto/ansioso/arrabbiato o agitato quando ti è stato proibito di fare sesso o masturbarti?
6. Ti è capitato di avere un'attività sessuale intensa che ha messo a rischio o a compromesso le tue relazioni sentimentali, il tuo lavoro, i tuoi risparmi, la tua salute, o i tuoi studi?
